# Immortalized common marmoset (*Callithrix jacchus*) hepatic progenitor cells possess bipotentiality in vitro and in vivo

**DOI:** 10.1038/s41421-018-0020-7

**Published:** 2018-05-15

**Authors:** Zhenglong Guo, Renwei Jing, Quan Rao, Ludi Zhang, Yimeng Gao, Fengyong Liu, Xin Wang, Lijian Hui, HaiFang Yin

**Affiliations:** 1 Department of Cell Biology, Tianjin Medical University, Qixiangtai Road, Heping District, Tianjin, 300070 China; 2 State Key Laboratory of Cell Biology, Institute of Biochemistry and Cell Biology, Shanghai Institutes for Biological Sciences, Chinese Academy of Sciences, Shanghai, 200031 China; 3 Department of Laboratory Animal, Tianjin Medical University, Tianjin, 300070 China; 4 Key Laboratory of National Education Ministry for Mammalian Reproductive Biology and Biotechnology, Inner Mongolia University, Hohhot, 010021 China; 5 Department of Laboratory Medicine and Pathology, Stem Cell Institute, University of Minnesota, Minneapolis, MN 55455 USA; 6 Hepatoscience Incorporation, 4062 Fabian Way, Palo Alto, CA 94303 USA

## Abstract

Common marmoset (*Callithrix jacchus*) is emerging as a clinically relevant nonhuman primate model for various diseases, but is hindered by the availability of marmoset cell lines, which are critical for understanding the disease pathogenesis and drug/toxicological screening prior to animal testing. Here we describe the generation of immortalized marmoset hepatic progenitor cells (MHPCs) by lentivirus-mediated transfer of the simian virus 40 large T antigen gene in fetal liver polygonal cells. MHPCs proliferate indefinitely in vitro without chromosomal alteration and telomere shortening. These cells possess hepatic progenitor cell-specific gene expression profiles with potential to differentiate into both hepatocytic and cholangiocytic lineages in vitro and in vivo and also can be genetically modified. Importantly, injected MHPCs repopulated the injured liver of fumarylacetoacetate hydrolase (*Fah*)-deficient mice with hepatocyte-like cells. MHPCs also engraft as cholangiocytes into bile ducts of 3,5-diethoxycarbonyl-1,4-dihydrocollidine (DDC)-induced bile ductular injured mice. MHPCs provide a tool to enable efficient derivation and genetic modification of both hepatocytes and cholangiocytes for use in disease modeling, tissue engineering, and drug screening.

## Introduction

The common marmoset (*Callithrix jacchus*) is a New World nonhuman primate, originally inhabiting in Brazil, South America, with some populations distributed in Australia and Africa^1^. Due to their small body size, ease of handling, shorter gestation period, and earlier sexual maturation as well as lower maintenance cost compared to Old World nonhuman primates, common marmosets are being used increasingly as one of the mainstream nonhuman primate animal models in biomedical research, including neuroscience, infectious diseases, reproductive biology, stem cell, and toxicological tests^2–7^. Previously, common marmosets were shown to serve as an ideal model system for hepatitis C virus (HCV)-related liver diseases in vivo and in vitro^8,9^. However, the understanding of the pathogenesis and development of effective treatments for liver diseases has been hindered by the availability of a suitable in vitro system.

Although numerous studies have demonstrated the feasibility of isolating primary hepatocytes or hepatic progenitor cells from human liver or other nonhuman primates such as rhesus monkey^10,11^, these cells can only undergo a few population doublings before losing their differentiation status and dying and lack the capability of indefinite expansion. Also this is particularly challenging experimentally because sacrificing nonhuman primates to obtain primary cells is both expensive and time-consuming, thus immortalized cell lines will greatly improve the functionality of nonhuman primates such as marmoset models. Epithelial liver stem cells from a cynomolgus monkey fetus could be immortalized^12^, suggesting that immortalized cell lines could be generated from nonhuman primate fetuses. Embryonic stem cell lines (ES) and induced pluripotent stem cell lines (ips) reported to be derived from common marmoset^13,14^ will be useful for understanding tissue differentiation in nonhuman primates, but are of limited use when it comes to diseases of specific tissues because of high costs and complicated differentiation protocols. Importantly, both ES- and ips-derived hepatocytes fail to expand in vitro indefinitely. Therefore, establishment of immortalized common marmoset hepatocytes or progenitor cells will provide a valuable tool for the development of effective treatments for liver diseases.

Here we describe the derivation of an immortalized hepatic progenitor cell line (marmoset hepatic progenitor cell; MHPC) from common marmoset fetal liver. Infection of fetal liver cells with simian virus 40 large T antigen (SV40T)-expressing lentivirus enables their indefinite proliferation in vitro without chromosomal alteration and telomere shortening. Examination on the gene expression profiles of this immortalized cell clone revealed a hepatic progenitor cell-specific gene expression, confirmed by semiquantitative reverse transcription-PCR and immunohistochemistry. MHPCs possess the capacities of self-renewal and ability to differentiate into both hepatocytes and cholangiocytes in vitro and in vivo. MHPCs are stable, expandable, and genetically editable, which hold great promise for the disease modeling and the development of effective treatments for liver diseases.

## Results

### Immortalized marmoset fetal liver cells are capable of self-renewal without chromosomal alteration and telomere shortening

**Fig. 1:**
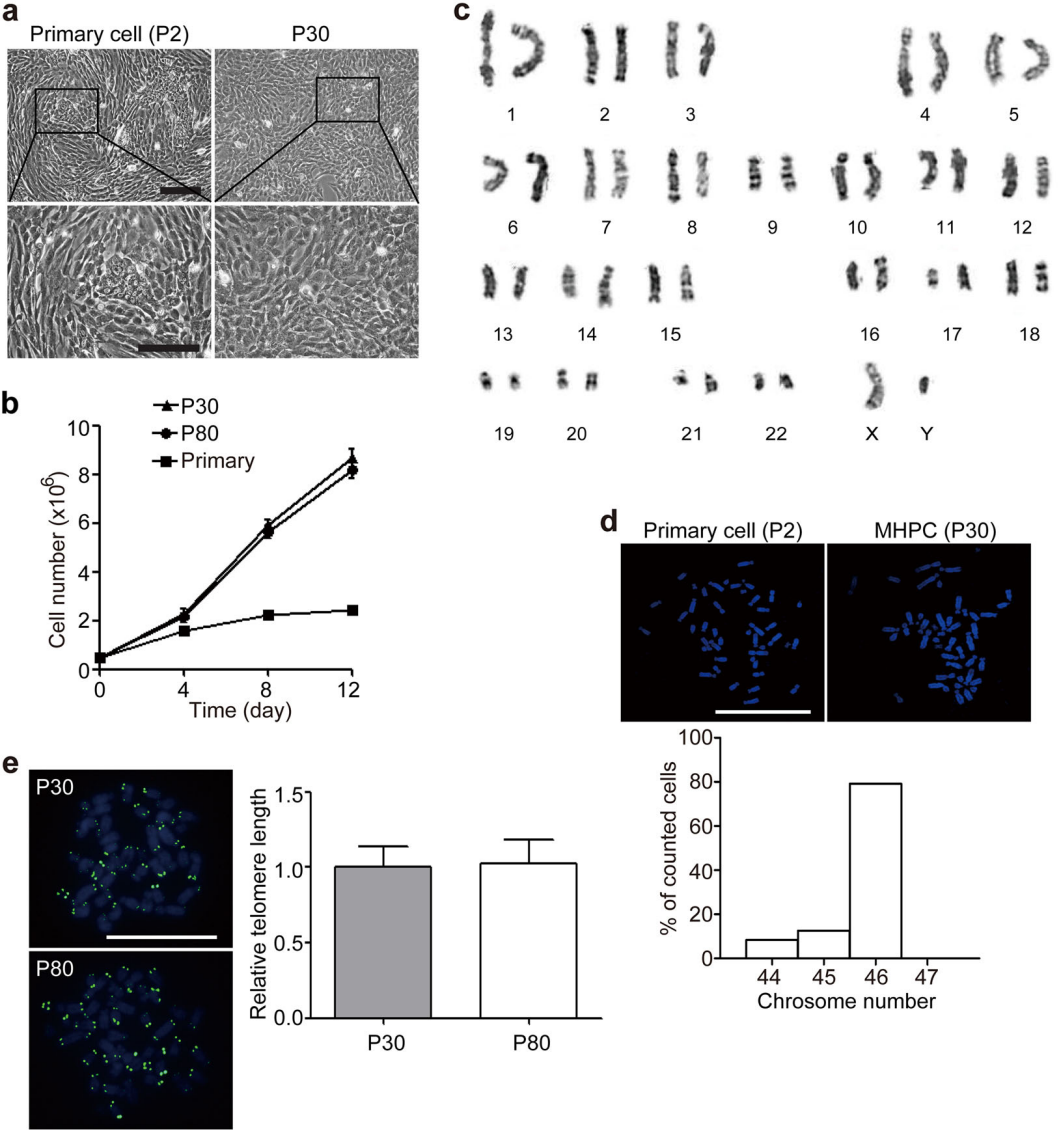
Establishment of immortalized marmoset fetal liver cells. **a** Morphology of immortalized marmoset fetal liver cell line MHPCs at passage 30 (scale bar = 100 µm). **b** Growth curve of MHPCs at passages 30 and 80, and isolated primary cells. Primary refers to isolated primary cells. Data represent mean ± sem (*n* = 3). The calculation was based on the online software http://www.doubling-time.com/compute.php. **c** G-band staining analysis of MHPCs at passage 30 showed a normal 46 XY karyotype. **d** Confocal microscopic images for DAPI staining of the choromosomes during mitosis. **e** Measurement of telomere lengths in MHPCs at passages 30 and 80 with Q-FISH. Data represent mean ± sem (*n* = 30)

A mixed population of fetal liver cells was derived from full-gestation aborted marmoset fetal liver after digestion with collagenase^15^. Approximately 70% of attached cells consisted of elongated spindle cells with dark nuclei, whereas about 30% of attached cells consisted of polygonal cells with epithelial-like morphology (Fig. 1a), which is a typical feature of hepatic progenitor cells. These cells were infected with lentivirus expressing SV40 large T antigen and enhanced green fluorescent protein (EGFP) daily for three times after one passage (Supplementary Figure S1a). Strong fluorescence signals were detected in mixed liver cells 1 week after three successive infections and EGFP-positive cell clones were sorted with fluorescence-activated cell sorting (FACS) and seeded in six-well plates with further dilution. Notably, EGFP signals became weaker with passaging (Supplementary Figure S1a), consistent with previous reports^16^. Six clones derived from a mixed population formed colonies and proliferated rapidly after 30 passages, though most cells changed their morphologies during infection and became more like elongated spindle cells (Supplementary Figure S1b). Among them, four clones showed the expression of both hepatocytic and cholangiocytic cell markers (Supplementary Figure S1c), and hepatic stem cell markers (sex-determining region Y box 9 gene—*Sox9*)^17^ and fetal hepatic progenitor markers (cytokeratin 7—*CK7*; Supplementary Figure S1d), indicating these clones are likely MHPCs. In contrast, clone 1 expressed all the markers, including hepatic stem cell marker (epithelial cell adhesion molecule—*EpCAM*) and fetal hepatic progenitor marker (α-fetoprotein—*AFP*; Supplementary Figure S1d), and was more epithelial-like compared to other clones and capable of indefinite proliferation with a mean population doubling time of 2.3 days (Fig. 1a, b). Little difference was detected in doubling time at passage 30 and passage 80 (Fig. 1b), suggesting that clone 1 retained the self-renewal capacity after multiple cell divisions in vitro. Karyotype analysis verified the normal 46 and XY karyotype of clone 1 after 30 population doublings as demonstrated by conventional Giesma banding (Fig. 1c). Examination on the number of chromosomes from 30 individual clones of clone 1 (passage 30) showed that approximately 82% of clones maintained 46 chromosomes (Fig. 1d) without telomere shortening (Fig. 1e), indicating that clone 1 can self-renew without chromosomal alteration and telomere shortening.

### MHPCs possess hepatic progenitor cell-specific expression pattern

**Fig. 2:**
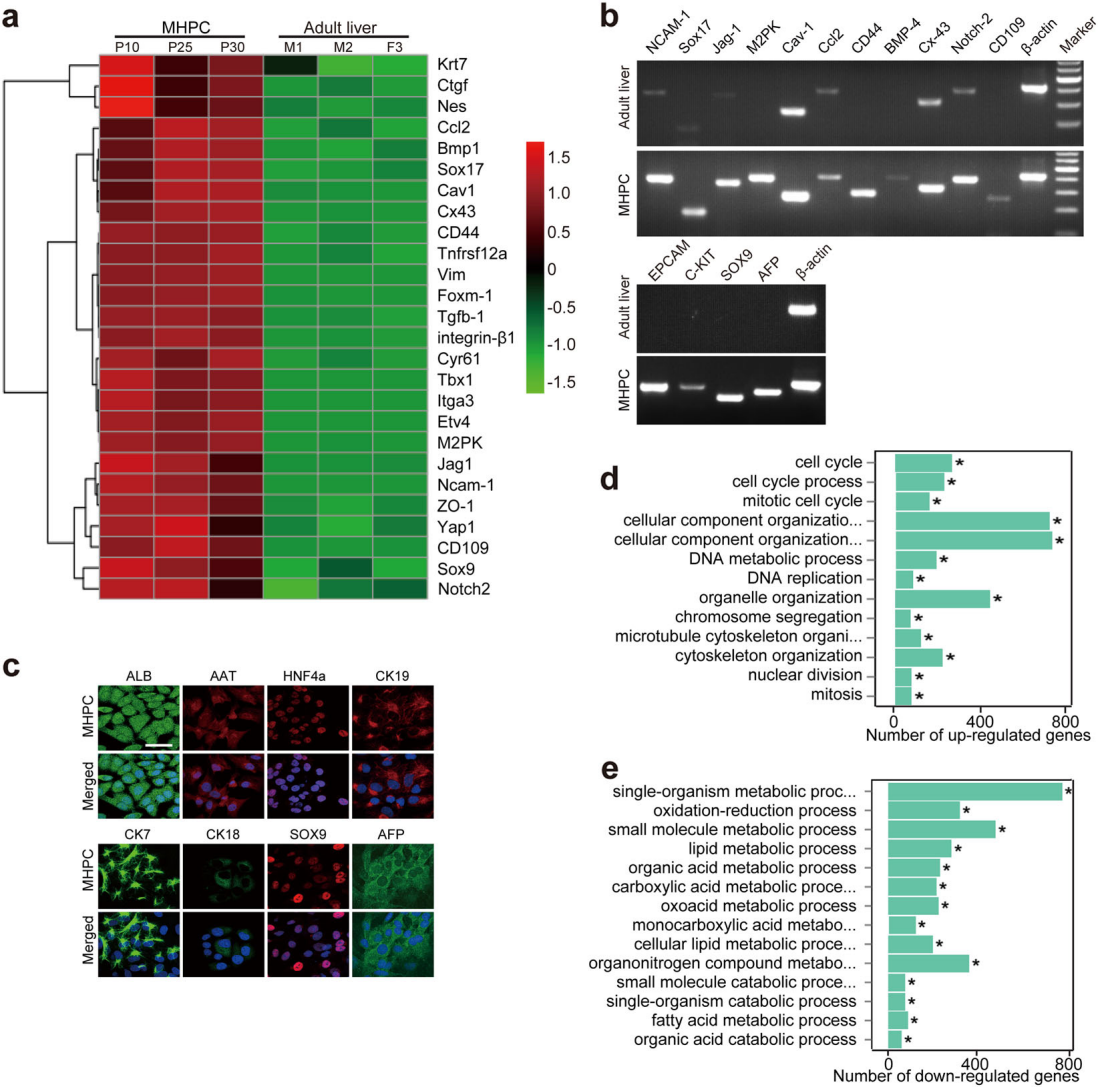
Lineage specification for MHPCs. **a** Hierarchial clustering analysis of global gene expression profiles to compare different passages of MHPCs with adult marmoset liver tissues. Passage 10, 25, and 30 MHPCs were used for RNA sequencing. M1 (male), M2, or F3 (female) refers to animal code. Expression levels (fold) are depicted in colors in which red represents upregulation and green means downregulation. **b** Semiquantitative RT-PCR analysis of hepatic progenitor cell markers for MHPCs at passage 30. Cav-1 refers to Caveolin-1; M2PK means tumor type M2 pyruvate kinase; Ccl2 chemokine (C-C motif) ligand 2; BMP4 bone morphogenetic protein 4; Cx-43 refers to Connexin 43. **c** Immunostaining of hepatic progenitor cell markers for MHPCs at passage 30. Nuclei was counterstained by DAPI (scale bar = 100 µm). **d** Gene set enrichment analysis (GSEA) of DNA replication and cell cycle-related genes for MHPCs at passage 30. DNA replication and cell cycle-related genes were significantly upregulated in MHPCs (passage 30) compared with adult marmoset liver tissues (*n* = 3, two-tailed *t*-test, *P < 0.1 × 10^−4^). **e** GSEA of cellular metabolism-related genes for MHPCs at passage 30. Cellular metabolism-related genes were significantly downregulated in MHPCs (passage 30) compared with adult marmoset liver tissues (*n* = 3, two-tailed *t*-test, *P < 0.1 × 10^-4^)

To specify the lineage of clone 1, we performed genome-wide expression profile analysis on different passages of clone 1 (passages 10, 25, and 30) and compared the global gene expression profiles with liver tissues derived from adult common marmosets of different ages. The expression profiling revealed that the upregulation of hepatic progenitor marker genes in clone 1, including *CD44*, *Sox9*, *Sox17*, and neural cell adhesion molecule 1 (*Ncam1*; Fig. 2a)^18^ compared to adult liver tissues, indicating that clone 1 is a MHPC line. The expression of hepatic progenitor-specific genes in MHPCs was further confirmed by semiquantitative RT-PCR (Fig. 2b) and immunocytochemistry (Fig. 2c). Consistently, gene set enrichment analysis demonstrated that the expression of DNA replication and cell cycle relevant genes was significantly elevated (Fig. 2d, *P < 0.1 × 10^−4^), whereas the mRNA levels of cellular metabolism-related genes were significantly decreased (Fig. 2e, *P < 0.1 × 10^−4^). Also pathways involved in stem cell development, including Wnt, Notch, and Hedgehog^19–21^ were also enriched in MHPCs when compared to adult liver tissues (Supplementary Figure S2). These data indicate that MHPCs are genuine hepatic progenitor cells.

### MHPCs show bipotentiality in vitro

**Fig. 3:**
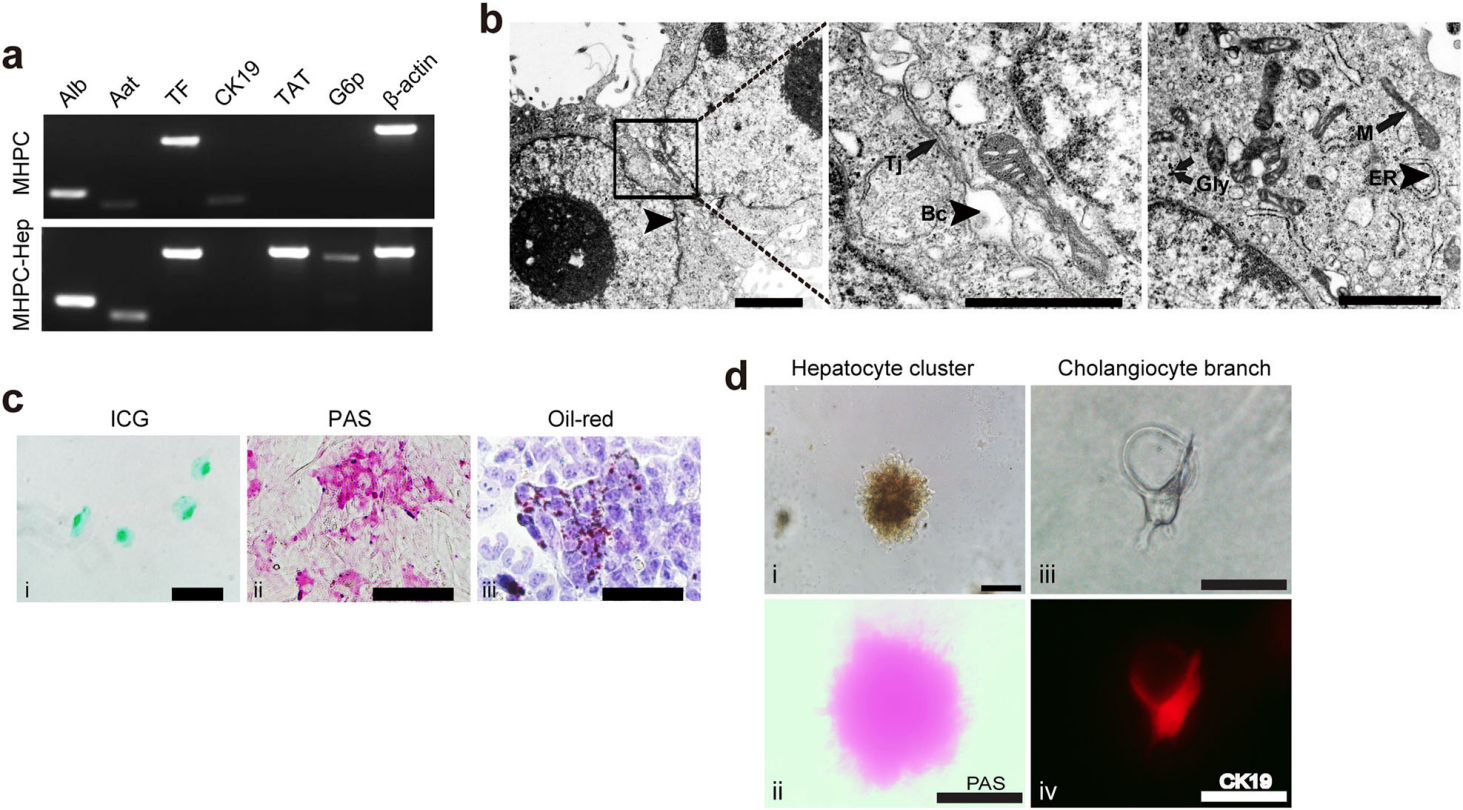
In vitro evaluation for the bipotency of MHPCs. **a** RT-PCR analysis of hepatocytic specific markers for differentiated MHPCs. MHPC-Hep refers to MHPC-derived hepatocytes. β-actin was used as a loading control. **b** Ultrastructure of MHPC-derived hepatocytes. Arrowheads indicate bile canaliculi (Bc) and endoplasmic reticulum (ER); arrows indicate tight junction (Tj), glycogen granules (Gly), and mitochondria (M) (scale bar = 1 µm). **c** In vitro functional evaluation of MHPC-derived hepatocytes, including (i) indocyanine green (ICG) uptake, (ii) PAS staining for glycogen storage, and (iii) Oil Red O staining for lipid accumulation. **d** In vitro bipotency of MHPCs, including (i) hepatic differentiation with 20 ng/ml OSM induction on 2D matrigel showed doughnut-like hepatocyte cluster morphology, and (ii) PAS staining for glycogen storage in MHPC-derived hepatocytes, (iii) branching structure of cholangiocytes formed by culturing in 3D type 1 collagen gel culture system, and (iv) CK19 staining (scale bar = 100 µm)

To investigate whether MHPCs possess the potency of hepatic progenitor cells to differentiate into mature hepatocytes and cholangiocytes in vitro, we cultured MHPCs in hepatic differentiation medium (HDM) with oncostatin M (OSM) and epidermal growth factor (EGF)^22^. Semiquantitative RT-PCR analysis revealed upregulation of mature hepatocyte-specific transcripts (albumin—*Alb*, α-1-anti-trypsin—*Aat*, tyrosine aminotransferase—*TAT*, transferring—*TF*, and glucose-6 phosphase—*G6p*) but not for cholangiocytic cell marker—*CK19* in MHPCs at 12 days after hepatic differentiation (Fig. 3a). Ultrastructural analysis of differentiated MHPCs with transmission electron microscopy indicated typical hepatocytic organelles such as mitochondria, lysosomes, glycogen granules, and bile canalicui in the intercellular space of adjacent cells (Fig. 3b). Moreover, differentiated MHPCs acquired mature hepatic functions, including indocyanine green (ICG) uptake (Fig. 3c-i), intracytoplasmic glycogen storage (Fig. 3c-ii), and lipid droplet generation (Fig. 3c-iii). When grown on two-dimensional Matrigel in HDM containing OSM and EGF for 6 days, MHPCs formed doughnut-like hepatocyte clusters with a diameter of 100 µm (Fig. 3d-i) and the hepatocytic differentiation status of MHPCs was confirmed by periodic-acid-Schiff (PAS) staining for glycogen storage (Fig. 3d-ii). When MHPCs were cultured in three-dimensional (3D) type I collagen gel culture system^23^, they readily differentiated into cholangiocyte-like cells with representative branching structures (Fig. 3d-iii) and expressed cholangiocyte marker CK19 after 9 days of induction (Fig. 3d-iv). Importantly, examination on lower (passage 10) and higher passages (50 and 80) of MHPCs revealed no difference in differentiation capacities among passages (Supplementary Figure S3a), though differentiated hepatocytes derived from primary fetal liver cells showed a weaker droplet formation ability compared to immortalized MHPCs (Supplementary Figure S3b). These findings support the conclusion that MHPCs possess the bipotentiality of differentiating into hepatocytes and cholangiocytes in vitro.

### Differentiated MHPCs are capable of detoxification and biliary secretion in vitro

**Fig. 4:**
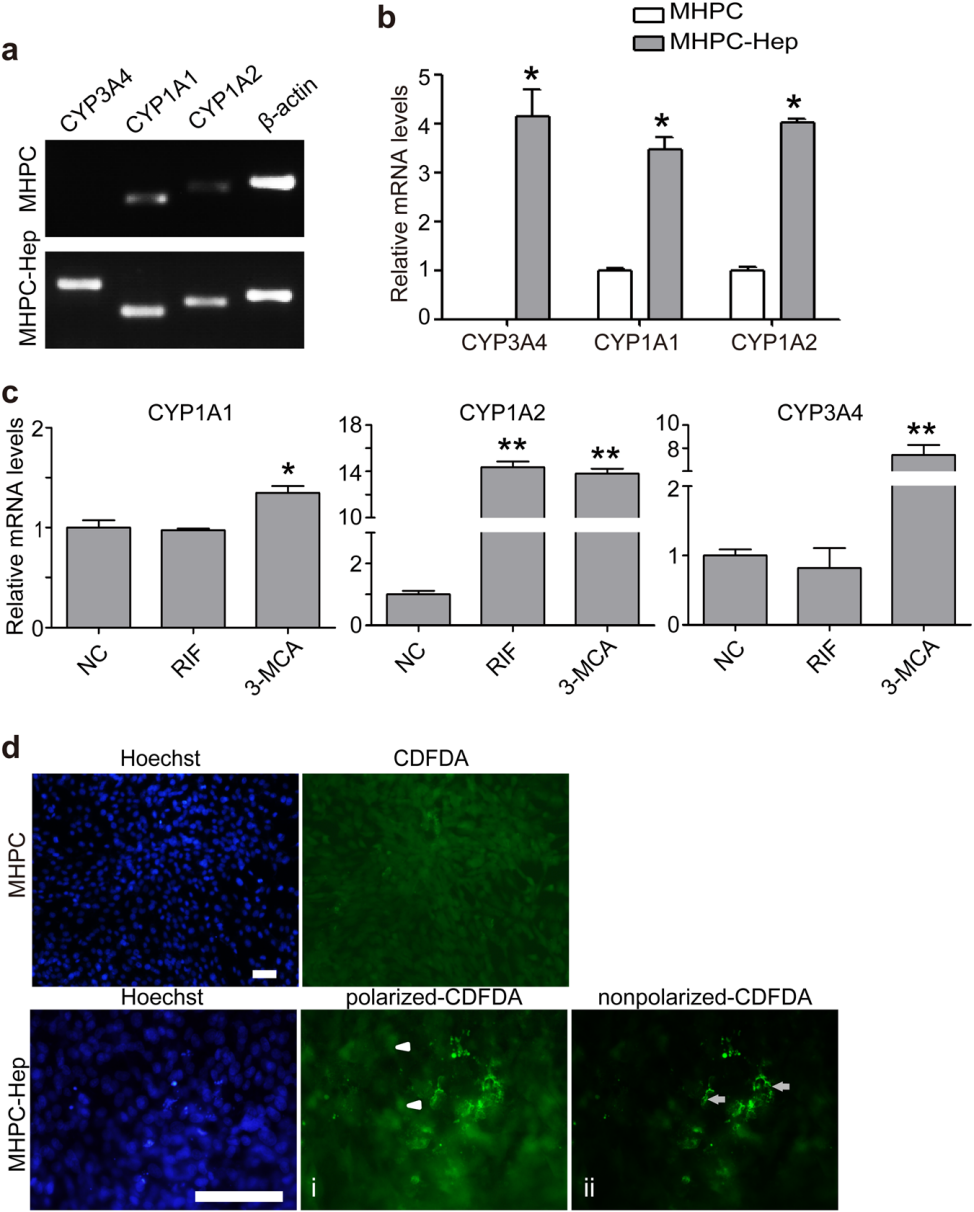
MHPC-derived hepatocytes possess CYP enzyme activities and biliary secretion function. **a** RT-PCR analysis for the levels of CYP enzyme expression in MHPC-derived hepatocytes. β-actin was used as a loading control. **b** Quantitative analysis of the mRNA levels of *CYP* genes by qPCR for MHPC-derived hepatocytes without inducer treatment. MHPC-Hep refers to MHPC-derived hepatocytes (*n* = 3, two-tailed *t*-test, *P < 0.05). **c** The mRNA levels of the CYP enzymes by qPCR, including CYP3A4, CYP1A1, and CYP1A2 in MHPC-derived hepatocytes after the induction with rifampicin (RIF) and 3-methylcholanthrene (3-MCA). Fold changes were normalized to the levels in the cells without induction, respectively (*n* = 3, two-tailed *t*-test, *P < 0.05; **P < 0.01). **d** CDFDA staining showed the accumulation of green CDF in functional bile canaliculi on the apical surface of MHPC-derived hepatocytes (scale bar = 100 µm). (i) Arrowheads point to CDFDA inside cells; (ii) arrows point to CDFDA around membrane

Drug detoxification is an important functional parameter for the functionality of mature hepatocytes, particularly for phase I drug metabolism, for which cytochrome P450 (CYP450) enzymes are largely responsible^24^. Examination on the expression of major CYP enzymes, including CYP3A4, CYP1A1, and CYP1A2 indicated that significantly higher levels of CYP3A4, CYP1A2, and CYP1A1 were detected in differentiated MHPCs than undifferentiated MHPCs (Fig. 4a, b). To analyze whether differentiated MHPCs were responsive to CYP inducers, we treated the cells with two commonly used chemical inducers, including 3-methylcholanthrene (3-MCA) and rifampicin (RIF), respectively, for 48 h. As expected, markedly increased mRNA expression levels of CYP3A4, CYP1A1, and CYP1A2 were induced by 3-MCA in differentiated MHPCs, though only a significantly higher level of CYP1A2 expression was detected in response to RIF induction (Fig. 4c), demonstrating that differentiated MHPCs have the capacity to detoxify drugs and can serve as an in vitro model system for studying drug metabolism. In addition, to evaluate the functional activity of epithelial surfaces on mature hepatocytes from differentiated MHPCs, which is likely lost during immortalization-induced epithelial-mesenchymal transition (EMT), we treated differentiated MHPCs with 5(6)-carboxy-2′, 7′-dichlorofluorescein diacetate (CDFDA), a functional assay for epithelial cell surface polarization^22^. Functionally polarized hepatocytes were defined by bright CDF-stained bile canaliculi as CDFDA can be hydrolyzed to fluorescent CDF and secreted to bile canaliculi. The results showed that bright fluorescence appeared in differentiated MHPCs with some punctuate signals localized inside cells (Fig. 4d-i, arrowheads), whereas others resided around membrane half an hour after incubation (Fig. 4d-ii, arrows), suggesting that differentiated MHPCs can absorb and hydrolyze CDFDA and thus secrete fluorescent CDF into bile canalicui. These results show that differentiated MHPCs enable drug detoxification and biliary secretion.

### MHPC transplantation alleviates Fah-deficiency-induced liver metabolic disease

**Fig. 5:**
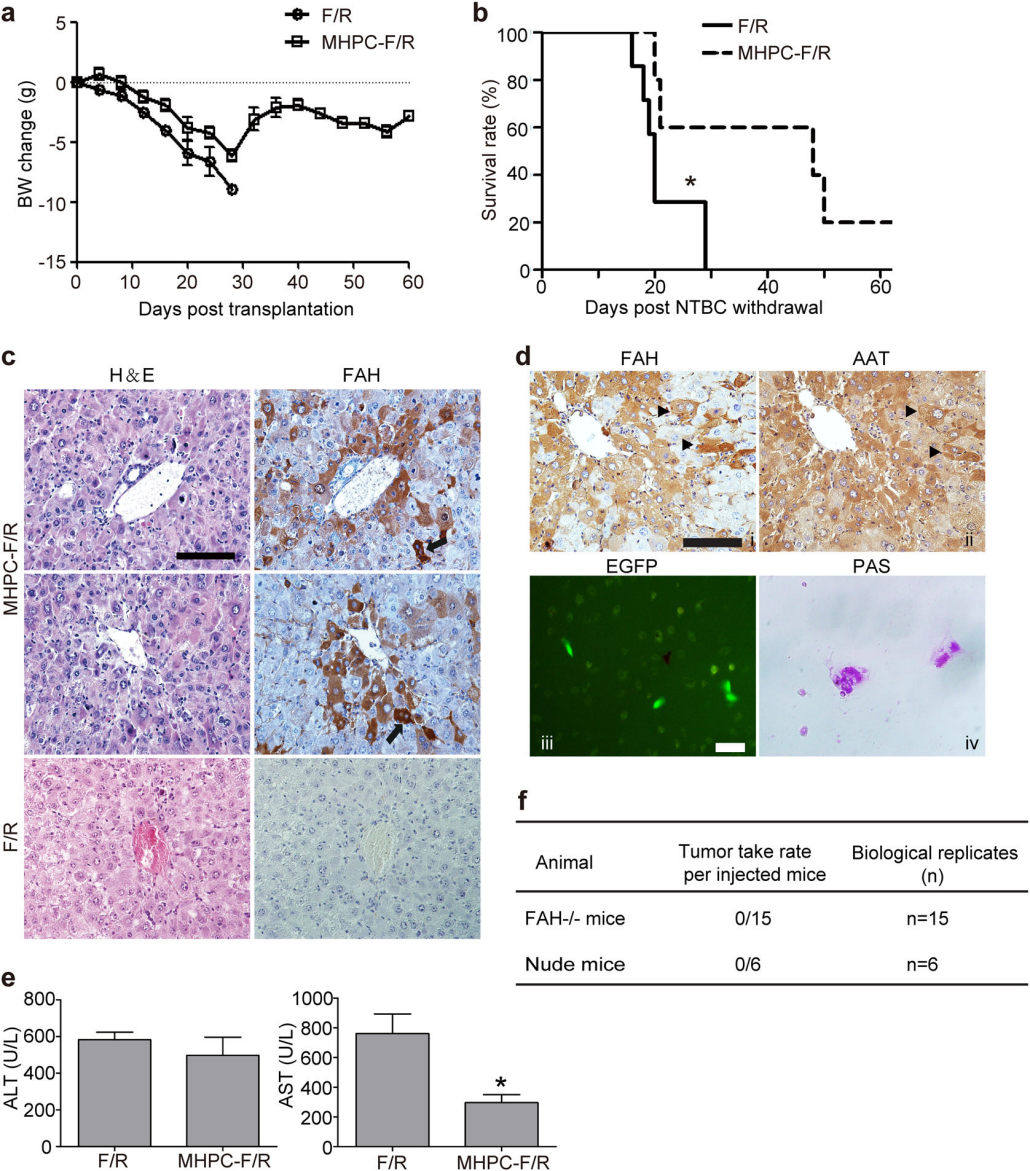
Engraftment of MHPCs into *Fah*^*−/−*^ (*F/R*) mice. **a** Body weight change of transplanted *F/R* mice. Body weight was measured twice every week in *F/R* mice after transplantation of 1 × 10^7^ MHPCs. Body weights at the indicated time were normalized to the weights prior to the transplantation. BW body weight. **b** Kaplan–Meier survival curves of MHPC-transplanted *F/R* mice (*n* = 5) and *F/R* mice that did not receive cells (*n* = 7) after NTBC withdrawal (two-tailed *t*-test, *P < 0.05). **c** Immunostaining for FAH in liver tissues from transplanted *F/R* mice. The results indicated the integration of MHPCs in *F/R* livers and showed normal hepatocyte morphology in H&E serial sections. Arrows indicate binucleated hepatocytes (scale bar = 100 µm). **d** Immunostaining for AAT in liver tissues and in vitro functional evaluation of isolated MHPC-derived hepatocytes from transplanted *F/R* mice. (i, ii) Serial staining for FAH and AAT in liver tissues from transplanted *F/R* mice. Arrows indicate the co-localization of FAH and AAT (scale bar = 100 µm). (iii) Isolated EGFP-positive cells showing glycogen storage stained by PAS (iv) (scale bar = 100 µm). **e** Levels of serum ALT and AST in transplanted and control *F/R* mice (*n* = 3, two-tailed *t*-test, *P < 0.05). The blood was collected prior to the experimental endpoints. **f** The tumorigenicity assay for MHPCs in vivo

Fumarylacetoacetate hydrolase-deficient (*Fah*^*−/−*^) mice defective in tyrosine metabolism require a supply of 2-(2-nitro-4-trifluoromethylbenzoyl)-1, 3-cyclohexanedione (NTBC) for survival, otherwise mice will die of liver failure. *Fah*^*−/−*^ mice can be rescued by transplantation of normal hepatocytes after NTBC withdrawal^25^, thus representing a good model to assess the functionality of mature hepatocytes. To investigate the potency of MHPCs to differentiate into mature hepatocytes in vivo, we intrasplenically injected EGFP-positive MHPCs (1 × 10^7^) into *Fah*^*−/−*^ mice 3 days after NTBC was withdrawn. EGFP was merely used as a reporter gene for tracing the cells after transplantation and the progenitor property of EGFP-infected MHPCs was verified by the expression of *Sox9* (Supplementary Figure S4a). Untreated *Fah*^*−/−*^ mice (*n* = 7) lost body weights gradually and died within 4 weeks after NTBC withdrawal (Fig. 5a, b). In contrast, although MHPC-transplanted *Fah*^*−/−*^ mice lost weight during the first 4 weeks post transplantation, they regained or stabilized body weights afterwards (Fig. 5a). Importantly, 60% of MHPC-transplanted *Fah*^*−/−*^ mice remained alive for at least 7 weeks without NTBC (Fig. 5b), suggesting that MHPC transplantation can extend the lifespan of *Fah*^*−/−*^ mice. Staining of liver tissues from MHPC-transplanted *Fah*^*−/−*^ mice indicated that Fah-positive cells derived from MHPCs comprised 1–7% of total hepatocytes in liver of *Fah*^*−/−*^ mice and all of the endogenous hepatocytes were Fah-negative (Fig. 5c, Supplementary Figure S4b). Strikingly, Fah-positive hepatocytes were found in multiple focal areas besides adjacent to central veins with some Fah-positive hepatocytes showing binucleated, a feature characteristic of mature hepatocytes^26^, similar to studies in mouse and human reported previously (Fig. 5c, d-i)^17,27^, suggesting that MHPCs can effectively engraft into the liver parenchyma of *Fah*^*−/−*^ mice. Serial staining of liver tissues from transplanted *Fah*^*−/−*^ mice demonstrated the co-localization of Fah- and AAT-positive cells (Fig. 5d-ii), indicating that Fah-positive cells are functional mature hepatocytes. As expected, Fah- and EGFP-postive hepatocytes were also found in spleen of *Fah*^*−/−*^ mice as reported for other species (Supplementary Figure S4c)^27^. To further confirm the in vivo function of differentiated MHPCs, we isolated EGFP-positive hepatocytes derived from MHPCs in transplanted *Fah*^*−/−*^ mice by FACS (Fig. 5d-iii) and cultured for 2 weeks in vitro. Glycogen accumulation was observed in isolated cells as demonstrated by PAS staining (Fig. 5d-iv), suggesting that isolated cells function as mature hepatocytes. Analysis of biochemical parameters for liver function including serum aspartate aminotransferase (AST) and alanine transaminase (ALT) revealed that the level of AST was significantly reduced, though to a much less extent with ALT (Fig. 5e). These findings demonstrate that MHPCs can repopulate *Fah*^*−/−*^ liver cells and attenuate liver injuries caused by Fah deficiency. Moreover, we found that MHPCs did not form tumors after transplantation in either *Fah*^*−/−*^ or immunodeficient nude mice (Fig. 5f).

### MHPCs contribute to bile duct proliferation in a 3, 5-diethoxycarbonyl-1, 4-dihydrocollidine-induced liver injury model

**Fig. 6:**
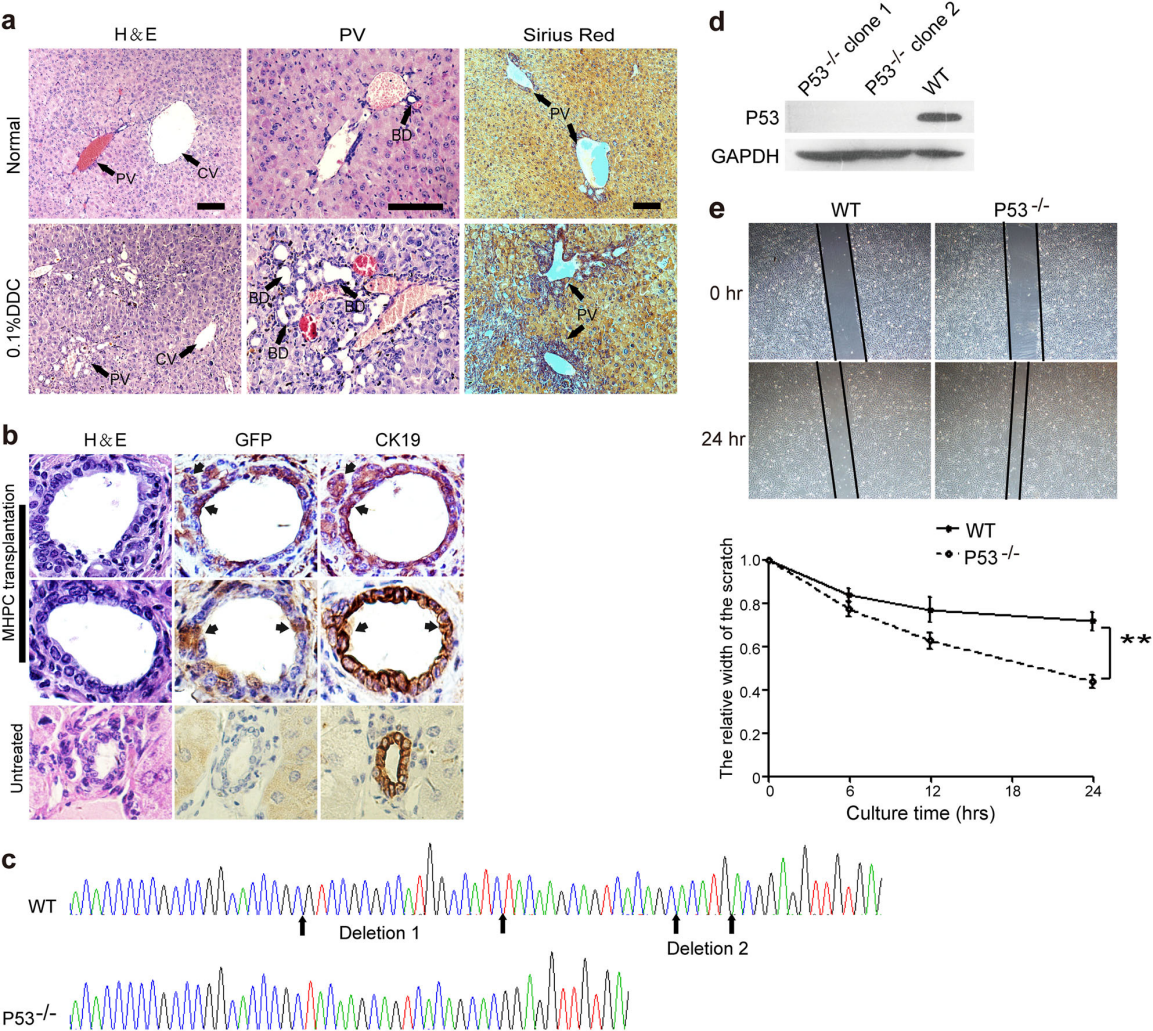
Engraftment of MHPCs into the DDC-induced injury mice and genetic modification of MHPCs. **a** H&E and Sirius Red staining of liver tissues from normal and 0.1% DDC-treated nude mice. Numerous small bile ducts (BD) appeared around the portal veins (PV) in the DDC liver (arrows). CV refers to central vein. **b** Serial immunostaining of GFP and CK19 in liver tissues from transplanted DDC-injured nude mice. The results showed the EGFP-positive MHPCs integrated in the large and small BD and expressed cholangiocyte marker CK19 (scale bar = 100 µm). **c** Sequence analysis of genetically modified MHPCs with CRISPR/Cas9 system for clone 1. Clones 1 and 2 showed identical deletions. WT refers to wild-type MHPCs. Two nonsense point mutations (A-C) and (C-G) were generated in the marmoset *p53* gene. **d** Western blot analysis for the expression of p53 in transfected MHPCs. GAPDH was used as a loading control. A unit of 130 µg total protein was loaded. **e** Wound-healing assay for examining the migration rate of p53^−/−^ MHPCs. A significantly faster migration rate was detected in p53^−/−^ MHPCs than WT MHPCs (*n* = 5, two-tailed *t*-test, **P < 0.01)

The 3, 5-diethoxycarbonyl-1, 4-dihydrocollidine (DDC)-fed mouse is a well-established model for hepatic progenitor cell activation and bile duct proliferation^28^. It was reported that in vivo DDC treatment can induce progenitor cells to spontaneously differentiate into cholangiocytes^29^. To determine whether MHPCs possess the ability to engraft into bile ducts and further differentiate into cholangiocytes in vivo. We first established a DDC-induced liver injury mouse model by feeding immunodeficient nude mice with 0.1% DDC (wt/wt). Bile duct proliferation was evident at 4 weeks after DDC induction as demonstrated by the increased number of small bile ducts and collagen deposition scattered throughout periportal areas and around the central and portal vein in the livers of DDC-induced mice (Fig. 6a). Importantly, all the bile duct proliferation were of nonhepatocyte origin revealed by the staining of CK19 (a cholangiocytic epithelial cell marker; Supplementary Figure S5a) and Sox9 (a progenitor cell marker; Supplementary Figure S5b)^30^ and the absence of AAT expression (a hepatocyte marker; Supplementary Figure S5c), suggesting that DDC-induced liver injury activates the endogenous progenitor cells to differentiate into cholangiocytes. EGFP-positive MHPCs (5 × 10^6^) were intrasplenically transplanted into DDC-induced mice (*n* = 8) and were found scattered within small and large bile ducts in the liver of two recipient mice 3 weeks post transplantation and comprised 10–20% of total biliary epithelial cells with the expression CK19 (Fig. 6b), demonstrating that MHPCs can be differentiated into cholangiocyte in vivo.

### MHPCs can be genetically modified with clustered regularly interspaced short palindromic repeat/Cas9

To examine the genetic modifiability of MHPCs, an important property for disease modeling, we targeted *p53*, a known tumor suppressor gene^31^, with the clustered regularly interspaced short palindromic repeat (CRISPR)/Cas9 system. Two different single-guide (sg) RNAs were designed (sgRNA-*p53*-1 and -2), targeting different sites of the marmoset *p53* exon 5, and introduced into MHPCs by lentivirus infection. Sequence analysis of two transfected MHPCs clones (clones 1 and 2) revealed two identical deletions in *p53* exon 5 (Fig. 6c) and further examination showed the absence of p53 protein as demonstrated by western blot (Fig. 6d), indicating that MHPCs can be readily genetically modified with CRISPR/Cas9 system. p53^−/−^ MHPCs showed no morphologic difference from normal MHPCs though a faster migration rate was observed with p53^−/−^ MHPCs than normal MHPCs as evidenced by the wound-healing assay (Fig. 6e).

## Discussion

Despite common marmosets are useful nonhuman primate models for medical research, particularly for HCV-related liver diseases, the unavailability of suitable in vitro model systems hinders the development of effective treatments for liver diseases. Herein we establish an immortalized MHPC showing an indefinite expansion capacity without chromosomal alteration and telomere shortening. MHPCs not only possess many characteristics of hepatic progenitor cells in self-renewal and gene expression profiles but also have bidirectional differentiation potential to both hepatocytic and cholangiocytic lineages in vitro and in vivo. Importantly, the ability to engraft and repopulate in *Fah*^*−*^^*/−*^ and immunodeficient nude mouse liver without tumorigenicity allows the development of preclinical protocols of allotransplantation in a primate model closely related to humans. In comparison to previous studies on immortalized liver stem cells from cynomolgus monkey fetus (namely IPFLS)^12^, MHPCs were derived from common marmoset, a New World nonhuman primate bearing different physiological features compared to Old World nonhuman primates such as cynomolgus monkey and also an ideal model system for HCV-related studies. The common marmoset remains a more cost-effective nonhuman primate model than the cynomolgus monkey, thus establishment of this cell line gives researchers more tools to work with before in vivo studies. Moreover, the potential of IPFLS to differentiate into biliary lineage and genetic modifiability remain to be determined. Thus, MHPCs would be useful to model hepatic progenitor cells in drug discovery and in understanding of the pathogenesis of human liver diseases.

In the current study, we employed a conventional approach for immortalizing mammalian cells by directly transfecting primary marmoset fetal liver cells with lentivirus expressing SV40 large T antigen^27^. In comparison to methods of staged differentiation to derive tissue-specific somatic cells from embryonic stem cells (ESCs) or induced pluripotent stem cells (iPSCs), the direct induction methods described here have advantages in saving time and materials. Notably, this approach also eliminates the possibility of contaminating cells types such as ESCs or iPSCs, which can result in the possibility of teratoma formation. However, the drawback of this virus infection-based immortalization method is the influence on cellular morphologies as reported earlier^16^, though spontaneous EMT acquisition during culture can also contribute to the morphological changes. Despite the acquisition of mesenchymal features of MHPCs during immortalization, MHPCs maintain the properties and potency of hepatic progenitor cells, including gene expression profiles and functionality, including epithelial cell surface polarization.

After induction in hepatocytic differentiation medium, differentiated MHPCs present the typical features of mature hepatocytes such as elevated expression of hepatocyte-specific gene expression, the presence of hepatocyte-specific organelles, and hepatocyte-related metabolic functions. Importantly, differentiated MHPCs show the capability of detoxification and biliary secretion, which is a key function of mature hepatocytes for detoxification^32^. An interesting observation from our study is that differentiated MHPCs respond remarkably well to CYP inducers such as 3-MCA, but only the level of CYP1A2 was significantly elevated by RIF induction in differentiated MHPCs. It was known that CYP induction is a time- and concentration-dependent process and the induction time and dose of different CYP inducers required varies in human and other species^33^. Therefore, we speculate that the unresponsiveness of CYP1A1 and CYP3A4 to RIF observed in differentiated MHPCs is likely due to the unfavorable conditions (i.e., induction time or concentrations), however the species differences cannot be excluded. Further investigations are warranted to optimize the conditions of RIF induction in MHPCs.

Previous studies showed that a survival rate of 30–50% is commonly observed after transplantation of human primary hepatocytes^34^, it is striking that the repopulation of MHPCs rescued around 60% of *Fah-deficient* mice in our study, suggesting that hepatic progenitor cells are probably more efficient than primary hepatocytes in repopulating liver cells in vivo and thus resulting in higher survival rate. Intriguingly, despite the high survival rate observed in *Fah-deficient* mice after MHPC transplantation, the level of serum ALT was only marginally decreased compared to untreated *Fah-deficient* mice. As it is known that ALT is more sensitive to hepatocytic injury, whereas the serum from untreated and treated *Fah-deficient* mice was harvested when the mice were dying. So it is reasonable that the level of serum ALT increased by the time of harvesting blood and tissues as the liver condition of MHPC-transplanted *Fah-deficient* mice deteriorated. Nevertheless, the significantly extended lifespan and expression of FAH and AAT observed in MHPC-transplanted *Fah-deficient* mice sufficiently proved the potency of MHPCs to repopulate liver cells in vivo and rescue *Fah-deficient* mice.

In summary, we establish an immortalized MHPC line and the cell line possesses the characteristic, gene expression pattern, bipotentiality of hepatic progenitor cells and can be genetically modified. These cells can be useful for studying the pathogenesis, disease modeling, treatments, and allotransplantation therapy for liver diseases.

## Materials and methods

### Cell isolation and culture

Marmoset fetal liver tissues were obtained from aborted marmoset fetus at 12–15 weeks of gestation. Fetal liver cells were isolated as previously described with minor modification^15^. Briefly, marmoset fetal liver tissues obtained were cut into small pieces and incubated in D-Hanks’ medium containing 0.05% collagenase type IV and 5 mM CaCl_2_ for 30–40 min at 37 °C. Dissociated cells were collected, filtered through 70 µm sterile gauze, and centrifuged at 500 × *g* for 5 min. The cell pellet was resuspended and seeded on six-well plates coated with type I collagen in medium Dulbecco’s modified Eagle’s medium (DMEM)/F12 (Invitrogen, USA) supplemented with 10% fetal bovine serum, 1% penicillin/streptomycin (Invitrogen), 0.1 mM 2-mercaptoethanol (Invitrogen), 10 ng/ml hepatocyte growth factor (HGF) and EGF (Invitrogen), 1× insulin-transferrin-selenium (ITS, Invitrogen), 1 × 10^7^ mol/l dexamethasone (Sigma, USA), and 10 ng/ml nicotinamide (Sigma). Cells were maintained at 37 °C in a 5% CO2 incubator with medium changed every other day. For passaging, confluent cultures were split 1:3 using 0.25% trypsin-EDTA for 2–5 min at 37 °C.

### Lentivirus preparation and infection

Lentivirus was prepared with three plasmids (40 µg in total), including the packaging plasmids psPAX2 and pM2D.G, and SV40 large T (kindly provided by Professor Lijian Hui, Shanghai Institutes for Biological Sciences, Chinese Academy of Sciences, Shanghai, China) or LentiCas9-Blast or LentiGuide-puro (purchased from Addgene), mixed in a ratio of 3:1:4 and 293FT cells were transiently transfected using calcium phosphate method. Viral supernatant from transfected 10 cm dish was collected every 24 h up to 2 days after the transfection. For SV40T-expressing lentivirus infection, fetal liver cells (passage 2, 5–10 × 10^5^) were seeded in 6-well plates and infected three times with 1.5 ml viral supernatant mixed with 0.5 ml fresh DMEM medium containing 1/1000 polybrene for 10 h and replaced with fresh DMEM medium for another 14 h prior to observation. For Cas9-expressing lentivirus infection, MHPCs (passage 50, 5 × 10^5^) were seeded in 12-well plates and infected with concentrated viral supernatant for 12 h as described above, followed by 2 µg/ml Blasticidin selection for 1 week. Cas9-expressing MHPCs were seeded in 6-well plates (1 × 10^6^) and transfected with two sgRNA-expressing lentivirus for 12 h, followed by 0.5 µg/ml puromycin selection for 2 weeks. Two sgRNAs targeting marmoset *p53* exon 5 were designed with the method reported previously^35^ and synthesized as follows: *p53* sgRNA-1: 5′-GCTTGTAGATGGCCATGGCG-3′; *p53* sgRNA-2: 5′-GCAGTCACAGCACATGACGG-3′. These sgRNAs were cloned into a LentiGuide-puro vector at BsmBI site as previously reported^36^.

### Animals

Six- to 8-week-old *Fah*^*−*^^*/−*^*Rag2*^*−/−*^ mice (kindly provided by Professor Xin Wang, University of Minnesota, MN, USA) and immunodeficient nude mice were used in all experiments (number was specified in the corresponding sections). The experiments were carried out in the animal unit (Tianjin Medical University, Tianjin, China), according to procedures authorized by the institutional ethical committee (Permit Number: SYXK 2009–0001).

### Karyotype and telomere analysis

The karyotype of MHPCs at passage 30 was analyzed. Briefly, MHPCs at passages 30 and 80 were incubated with 10 µg/ml mocodazole (Sigma) in fresh culture medium for 6 h to enrich arrested metaphases, followed by treatment with 0.75 M KCl for 40 min at 37 °C, then fixed with cold methanol:acetic acid (3:1) and spread onto a clean glass slide. For G-band analysis, cells were fixed in 4% paraformaldehyde (PFA) and stained with Giemsa solution, followed by analysis with standard protocols^37^. Chromosome numbers was counted in at least 30 individual cells with fluorescence microscope (Olympus FV1000, Olympus, Japan) after staining with 4′,6-diamidino-2-phenylindole (DAPI). Telomere length of MHPCs was estimated by quantitative fluorescence in situ hybridization^38^. Slides were denatured and hybridized with 0.5 µg/ml fluorescein isothiocyanate-labeled peptide nucleic acid probe (Panagene, Korea), and were counterstained with 0.5 µg/ml DAPI. Chromosomes and telomeres were imaged with fluorescence confocal microscope. Data were analyzed using the online software “Telometer: Software for Telomere Counting” (http://demarzolab.pathology.jhmi.edu/telometer/). The relative telomere length was calculated based on the ratio of the fluorescence intensity sum of all telomere pixels for a given chromosome (proportional to telomere length) to the intensity sum of DAPI pixels for the corresponding chromosome. The mean value for 46 chromosomes for each cell was obtained. For quantitative analysis of telomere length, at least 30 individual cells were measured.

### RNA sequencing

Total RNA (3 µg/sample) extracted from three adult marmoset livers (3, 6, and 12 years old, respectively) and from MHPC cells at different passages (passges 10, 25, and 30, respectively) was used as input material. Sequencing libraries were generated using NEBNext^®^ Ultra™ RNA Library Prep Kit for Illumina^®^ (NEB, USA) per manufacturer’s instructions and index codes were added to attribute sequences to each sample. The clustering of the index-coded samples was performed on a cBot Cluster Generation System using TruSeq PE Cluster Kit v3-cBot-HS (Illumia, USA) according to the manufacturer’s instructions. After cluster generation, the library preparations were sequenced on an Illumina Hiseq 2500 platform and 100/50 bp single-end reads were generated. Differential expression analysis of two conditions/groups (two biological replicates per condition) was performed using the DESeq R package (1.10.1). DESeq provides statistical routines for determining differential expression in digital gene expression data using a model based on the negative binomial distribution. The resulting *P*-values were adjusted using the Benjamini and Hochberg’s approach for controlling the false discovery rate. Genes with an adjusted *P*-value < 0.05 found by DESeq were assigned as differentially expressed. Gene Ontology (GO) enrichment analysis of differentially expressed genes was implemented by the GOseq R package, in which gene length bias was corrected. GO terms with corrected *P* < 0.05 were considered significantly enriched by differentially expressed genes. Clustered heat-maps were produced by cluster 3.0 software. Original data were uploaded to Gene Expression Omnibus database with accession number: GSE99168.

### Reverse transcription-PCR

Total RNA was extracted from cells by Trizol (Invitrogen) and 1 µg RNA as template was reverse transcribed into cDNA with Transcriptor first strand cDNA synthesis Kit (Roche, Switzerland) per the manufacturer’s instructions. Semiquantitative PCR was performed with Taq polymerase. Quantitative real-time PCR (qPCR) was performed with SYBR Premix Ex Taq (TaKaRa, Japan) on ABI StepOnePlus real-time PCR system (Applied Biosystems, USA). Primers used for the study were listed in Supplementary Table S1. All qPCR data were performed with at least three repeats. The PCR products were confirmed by proper melting curves and an agarose gel electrophoresis.

### Immunocytochemistry and immunohistochemistry

For immunocytochemistry, cells were fixed with 4% PFA for 30 min at 4 °C, then permeabilized with 0.1% Triton X-100 for 30 min and blocked with 5% goat serum for 2 h at room temperature (RT). Subsequently, cells were incubated with primary rabbit monoclonal antibodies, including Albumin, CK19, CK7 (Abcam, UK, 1:200), CK18 (1:100), and Sox9 (1:250, Abcam), and α-1-antitrypsin (rabbit polyclonal antibody, Abcam, 1:200), and mouse monoclonal antibodies, including AFP and hepatocyte nuclear factor 4α (Abcam, 1:200) at 4 °C overnight, and then incubated with goat anti-rabbit Alexa-488, donkey anti-rabbit Alexa-594, or goat anti-mouse Alexa-488 secondary antibodies were used at a dilution of 1:200 in phosphate-buffered saline with Tween 20 (PBST) for 1 h at RT, followed by counterstaining with DAPI for 15 min at RT. For immunohistochemistry, tissues were fixed in Bouin’s solution (Sigma) and embedded with paraffin. Paraffin sections (5–7 μm thick) were prepared, slides were treated with 3% hydrogen peroxide for 15 min, antigens were retrieved by incubating the section in 2.94 g/l citrate buffer (pH = 6.0) with microwave. Slides were blocked in 10% goat serum in 1% bovine serum albumin-PBS for 2 h, and incubated with the primary rabbit monoclonal antibodies, including GFP, CK19 (Abcam, 1:200), and monoclonal rabbit antibodies FAH (Cell Lab Tech, US, 1:3000), α-1-antitrypsin (Abcam, 1:200) overnight at 4 °C, and then incubated with either goat anti-rabbit or anti-mouse horseradish peroxidase-labeled secondary antibodies were used at a dilution of 1:5000 in PBST for 1 h at RT, followed by 3,3′-diaminobenzidine staining.

### Histology

Routine hematoxylin and eosin staining was applied. For sirius red staining, liver tissues from DDC-fed and wild-type nude mice were fixed with 4% PFA and embedded in paraffin. Paraffin sections (5–7 μm thick) were prepared and slides were stained with Sirius red solution (Sigma) for 20 min, and then washed with PBS for three times, followed by staining with hematoxylin for 10 min. Slides were observed with conventional microscopy (Olympus FV1000, Olympus) for pathological evaluation.

### PAS, ICG, and CDFDA uptake assays and Oil Red O staining

Cells were stained by PAS kit (Sigma) per the manufacturer’s instructions. For ICG (Sigma) uptake assay, cells were incubated with DMEM containing 1 mg/ml ICG at 37 °C for 1 h, followed by washing with PBS three times. ICG release was examined 6 h later. For Oil Red O staining, confluent cells were fixed with 4% PFA at 4 °C for 30 min, followed by Oil Red O (Sigma) staining for 15 min, and then washed with PBS and stained with hematoxylin (Sigma) for 30 s. For polarization assay, cells were incubated with 10 µg/ml CDFDA (Sigma) and 1 µg/ml Hoechst 33342 for 30 min at 37 °C, followed by washing with ice-cold PBS and observed with fluorescence confocal microscope (Olympus FV1000, Olympus).

### In vitro differentiation

For the generation of hepatocytes, 1 ml Matrigel (BD, USA) was poured into six-well Ultra Low Cluster Plates (Corning Costar, USA) and then was placed at 37 °C for 30 min to form the gel. Cells (1 × 10^5^) were suspended in HDM (DMEM/F12 medium supplemented with 10% fetal bovine serum (FBS; Invitrogen), 20 ng/ml OSM (R&D, USA), 20 ng/ml EGF (Gbico, USA), 10 ng/ml nicotinamide, 0.1 mmol/l l-ascorbic acid and 1 × 10^7^ mol/l dexamethasone (Sigma). For cholangiocyte differentiation, 3D culture system using collagen type 1 (BD) was prepared as previously described^23^. Briefly, 800 µl collagen type 1, 100 µl 10 × PBS, 20 µl 1 N NaOH, and 80 µl H2O were mixed on ice. This mixture was further mixed with an equal volume of 1 × 10^5^ cells suspended in cholangiocytic differentiation medium (DMEM/F12 medium supplemented with 10% FBS, 20 ng/ml HGF, 1× ITS (Invitrogen).

### CYP450 induction

To evaluate CYP450 induction, MHPC-derived hepatocytes were incubated with 3-MCA (25 µM), RIF (25 µM), or dimethylsulfoxide (DMSO) dissolved in culture medium for 48 h. Total RNA was extracted to measure the level of CYP enzymes, including CYP3A4, CYP1A1, and CYP1A2, in response to chemical inducers by qPCR. DMSO was used as a control for the normalization of CYP expression.

### Mice breeding and cell transplantation

To suppress the immune response in *Fah*^*−/−*^*Rag2*^*−/−*^ mice, 40 µl anti-mouse asialo-GM1 (1 mg/ml; Wako, Japan) was intraperitoneally injected into *F/R* mice 24 h before transplantation. After transplantation, mice were administrated with 7.5 mg/l FK506 (Astellas, Japan) in the drinking water every day and GM1 was given every 7 days. For nude mice, 7.5 mg/l FK506 was given in the drinking water post transplantation. *Fah*^*−/−*^*Rag2*^*−/−*^ mice were maintained with 7.5 mg/l NTBC in the drinking water. For cell transplantation, similar methods were used as described previously^27^. Briefly, NTBC was reduced to 3.75 mg/l for 3 days and withdrawn for another 3 days prior to transplantation. EGFP-expressing MHPCs (1 × 10^7^) in 120 µl Dulbecco‘s Phosphate Buffered Saline (DPBS) were injected intrasplenically into the *Fah*^*−/−*^*Rag2*^*−/−*^ mice without NTBC. Body weight was monitored twice a week after transplantation. For the control group (*n* = 7), blood and liver samples were collected when 30% body weight was lost. For transplanted group (*n* = 5), blood and liver samples were collected 8–10 weeks post transplantation. For DDC-induced liver injury mice, nude mice (*n* = 7) were put on a diet containing 0.1% DDC (wt/wt) for 3 days, then mice were transplanted intrasplenically with 5 × 10^6^ EGFP-expressing MHPCs in 60 µl DPBS. After 4–5 weeks, mice were sacrificed and liver samples were collected.

### Tumorigenicity assay

MHPCs (1 × 10^6^) were subcutaneously injected into the flanks of 6- to 8-week-old nude mice (*n* = 6), animals were examined twice a week to see the formation of tumors during 3-month period.

### Serum enzyme measurements

Mouse blood was taken immediately after cervical dislocation and centrifuged at 1500 rpm for 3 min. Serum was separated and stored at −80 °C. Analysis of levels of serum AST and ALT were performed by the clinical laboratory (Tianjin Metabolic Disease Hospital, Tianjin Medical University).

### Wound-healing assay

MHPCs and p53^−/−^ MHPCs (1 × 10^6^) were seeded in six-well plates for 24 h and then cultured in serum-deprived growth medium for 12 h. Cells were scratched with a sterile pipette tip and cultured in DMEM containing 2% FBS for another 24 h. The relative width of the scratch was measured with a ruler and five areas were randomly selected.

### Statistical analysis

All data are reported as mean values ± sem. Statistical differences between treatment and control groups were evaluated by Sigma Stat (Systat Software, London, UK). Both parametric and non-parametric analyses were applied, in which the Mann–Whitney rank sum test (Mann–Whitney *U*-test) was used for samples on a non-normal distribution, whereas a two-tailed *t*-test was performed for samples with a normal distribution.

## Electronic supplementary material

Supplementary Materials(DOCX 9855 kb)
