# Supplementary material for: Immortalized common marmoset (Callithrix jacchus) hepatic progenitor cells possess bipotentiality in vitro and in vivo
Source: Cell Discov. 2018 May 15;4:23. doi: 10.1038/s41421-018-0020-7 (PMC5951880; doi:10.1038/s41421-018-0020-7)
Supplement: Supplementary file 1 — Supplementary Materials(DOCX 9855 kb) [file 41421_2018_20_MOESM1_ESM.docx]

**Supplementary Tables**

**Supplementary Table S1 Primers used in the study**

| Gene | Forward (5’-3’) | Reverse (5’-3’) |
| --- | --- | --- |
| Alb | GTGGGCAGCAAATGTTGTAA | TCATCGACTTCCAGAGCAGA |
| Aat | GTGGGCAGCAAATGTTGTAA | TCATCGACTTCCAGAGCAGA |
| TF | GAAGGACCTGCTGTTTAAGG | CTCCATCCAAGCTCATGGC |
| G6p | TAGCAGAGCAATCACCACCA | GCTTTATCAGTGGCACGGAG |
| Hnf1a | CACCGATGCCGTTAGAAGTG | CCTGTGTTGGTGAATGTGGG |
| Hnf4a | CAGGGTCTTGGGTGGGTATT | AACTGCCGTGTTCATTTCCC |
| Ck19 | CCGCGACTACAGCCACTACT | GAGCCTGTTCCGTCTCAAAC |
| Ck7 | CAGGATGTGGTGGAGGACTT | GGTTCTTGATGGCGTCGATC |
| Ck18 | ACGAGAAGGAGACCATGCAA | GTCATCAGCAGCAAGACGAG |
| Epcam | CTTTAAGGCCAAGCAGTGCA | TCCAGATCCAGTTGTTCCCC |
| c-kit | ACCAACACCGGCAAATACAC | GCTTGCTTTGGACACAGACA |
| Sox9 | GAAGAGAGAGAGGACCAGCC | CACACCGAATCCCAACGATTC |
| Afp | TGGCCTCTTCCGGAAACTAG | GAGAATGCGGGAGGGACATA |
| β-actin | TCCTGACCCTGAAGTACCCC | GTGGTGGTGAAGCTGTAGCC |
| Cyp3a4 | GCAGCTGAAAGGAAGACTCG | AAAGGCCTCCGGTTAGTTGAA |
| Cyp1a1 | TTTGGCCAGCTCTATGACCA | CGTTCTCATCCAGCTGCTTC |
| Cyp1a2 | TGGCACTGACGAAGATGAGT | GCCTCCTTGCTTACATGCTC |
| TAT | TGAGAACGACGTGGAGTTCA | AAGGGAGAATCTGGGGTGTG |
| Ncam-1 | GTCGTCACTGACCCTGAAGA | TCCAATGACTCCTGCCCAAT |
| Sox17 | CTCCGGATGTTTGCTGCTTT | CTGATCCTTCGCTTGTCACG |
| Jag-1 | TAACCGTGGCTTGGATCTGT | GGATGTTTTGTCGGTGTGCT |
| M2pk | ATGGCTGACACATTCCTGGA | GGCGTTATCCAGCGTGATTT |
| Cav-1 | GAAAACGGAGGCCATTGTGT | CAGGCAGCGTTCACTATGAC |
| Ccl2 | GCAGCAAGTGTCCCAAAGAA | CCCAGGGGTAGAATTGCAGT |
| Cd44 | ACCATTTCAACCACACCACG | AGTTGCCTGGATTGTGCTTG |
| Bmp-4 | TGGGCTGGAATGACTGGATT | GTAGTGTGTGGGTGAGTGGA |
| Cx-43 | AAAAGAGATCCCTGCCCACA | TCGCCAGTAACCAGCTTGTA |
| Notch-2 | TGCCCACCATGTACCAGATT | ATGTTGTTGTGTGGTGGCTC |
| Cd109 | CTGCAAGCCTTCCAACCATT | CAGATGTGTCGGCTTGATGG |

**Supplementary Figures**

**Supplementary Figure 1**

**
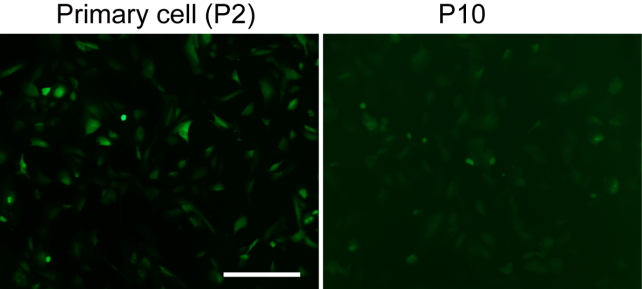
a**

**b**


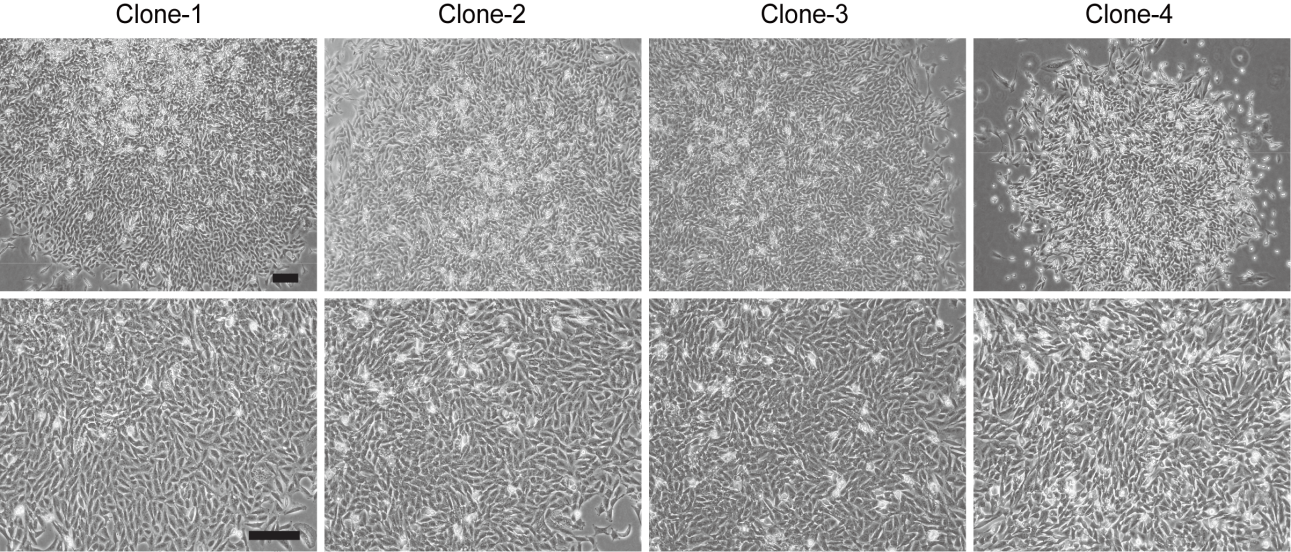


**
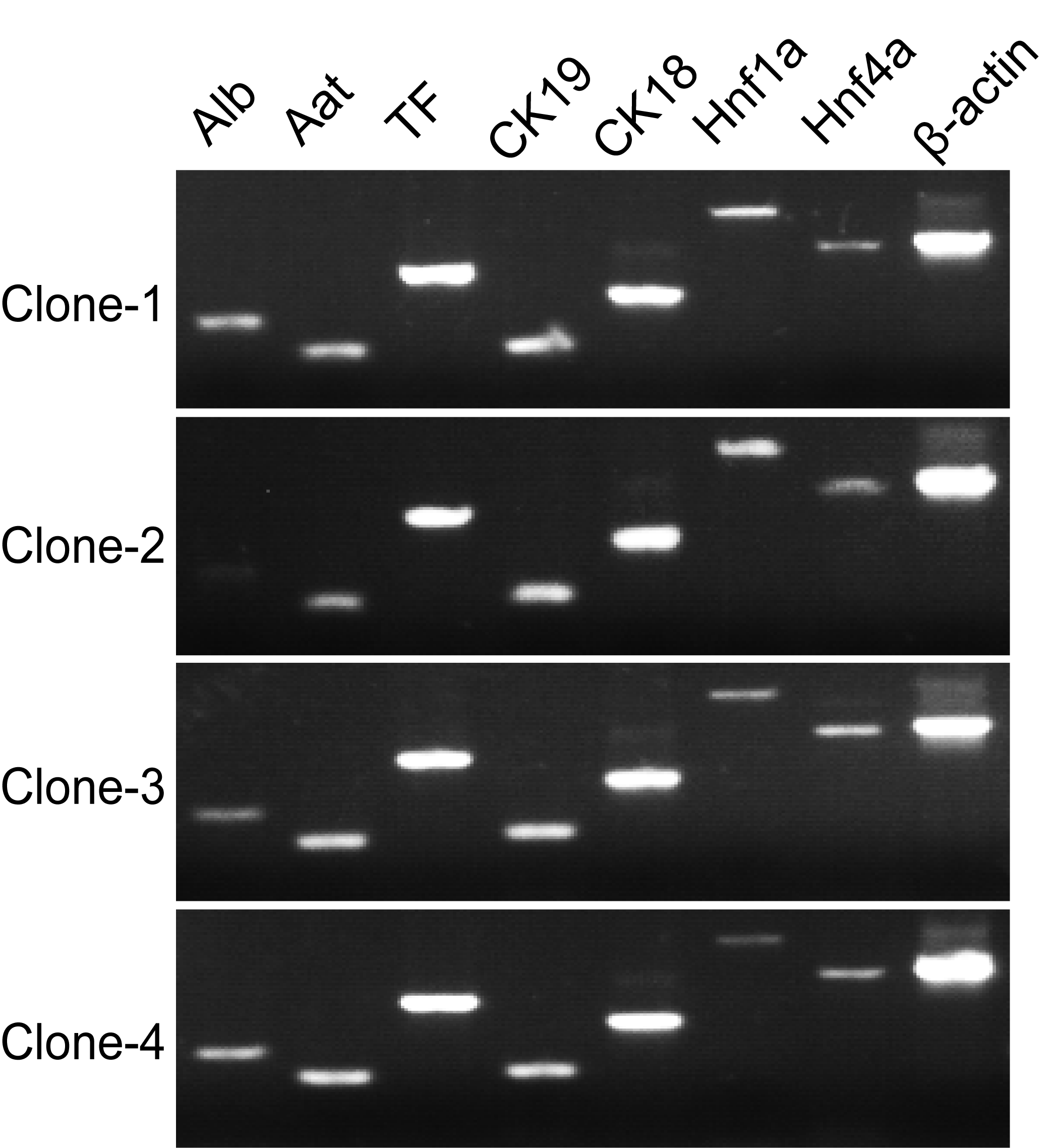
c**

**
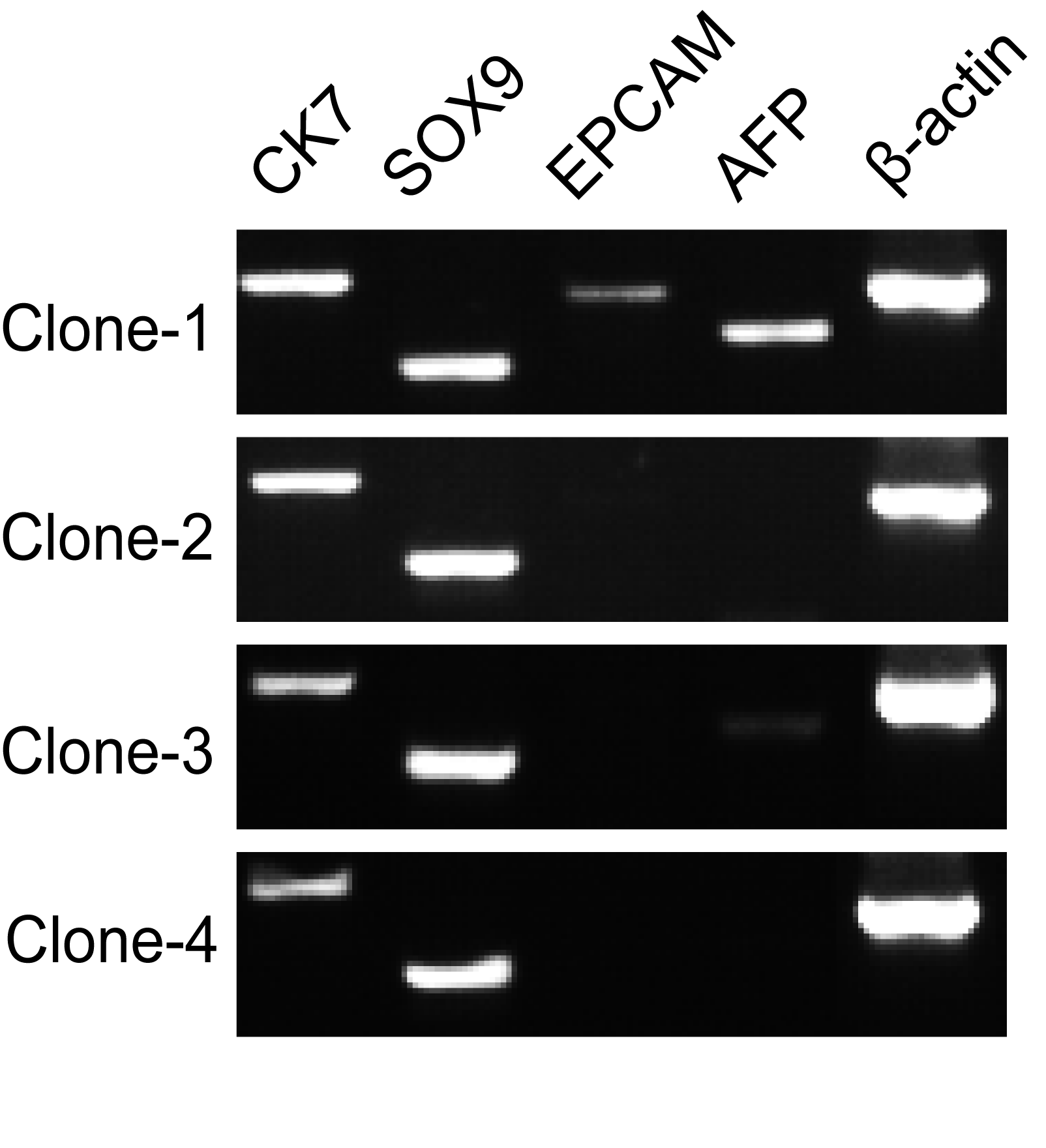
d**

**Supplementary Figure 1. Transfection and characterization of marmoset fetal liver cell clones.** (**a**) EGFP expression in primary cells and immortalized fetal liver cells at passage 10 (scale bar=100 µm). (**b**) Morphology of different immortalized fetal liver cell clones (passage 30). (**c**) RT-PCR analysis of hepatocytic and cholangiocytic cell markers in different cell clones (passage 30). (**d**) RT-PCR analysis of hepatic progenitor cell markers in different cell clones (passage 30). β -actin was used as a loading control.

**Supplementary Figure 2**


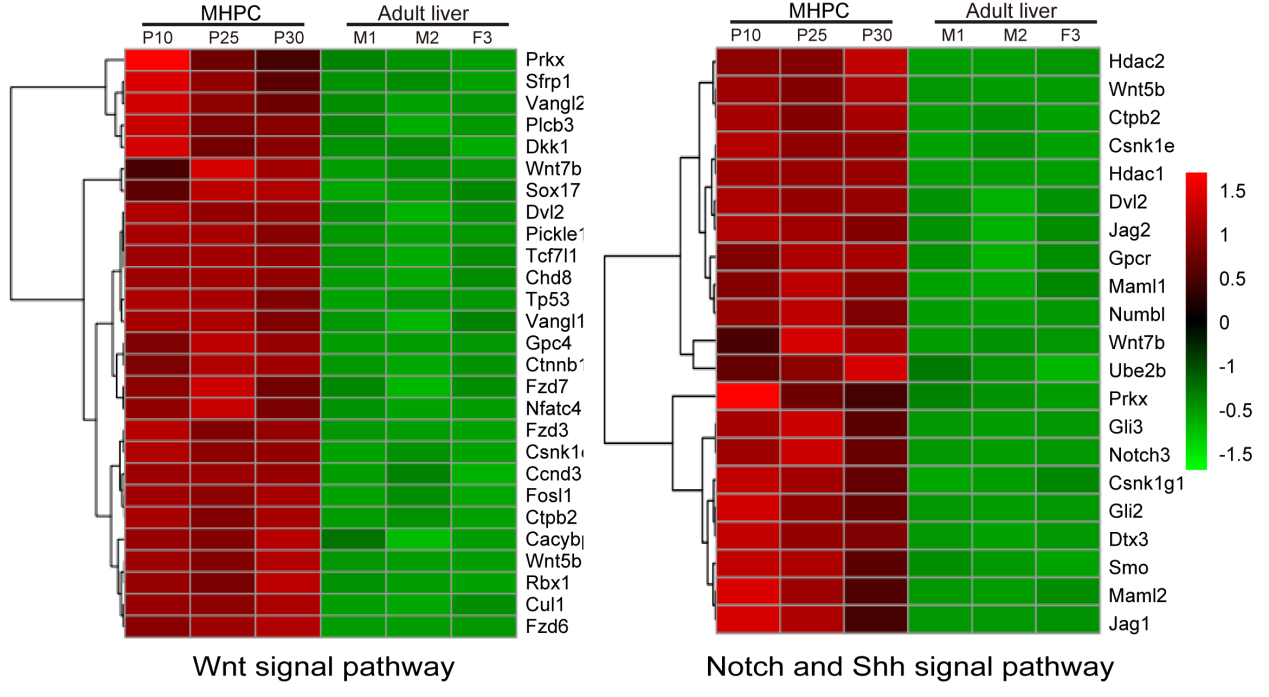


**Supplementary Figure 2.** Hierarchial clustering analysis of Wnt, Notch and Shh signal pathway related gene expression profiles to compare different passages of MHPCs with adult marmoset liver tissues. Passage 10, 25 and 30 MHPCs were used for RNA sequencing. M1 (male), M2 or F3 (female) refers to animal code.

**Supplementary Figure 3**

**
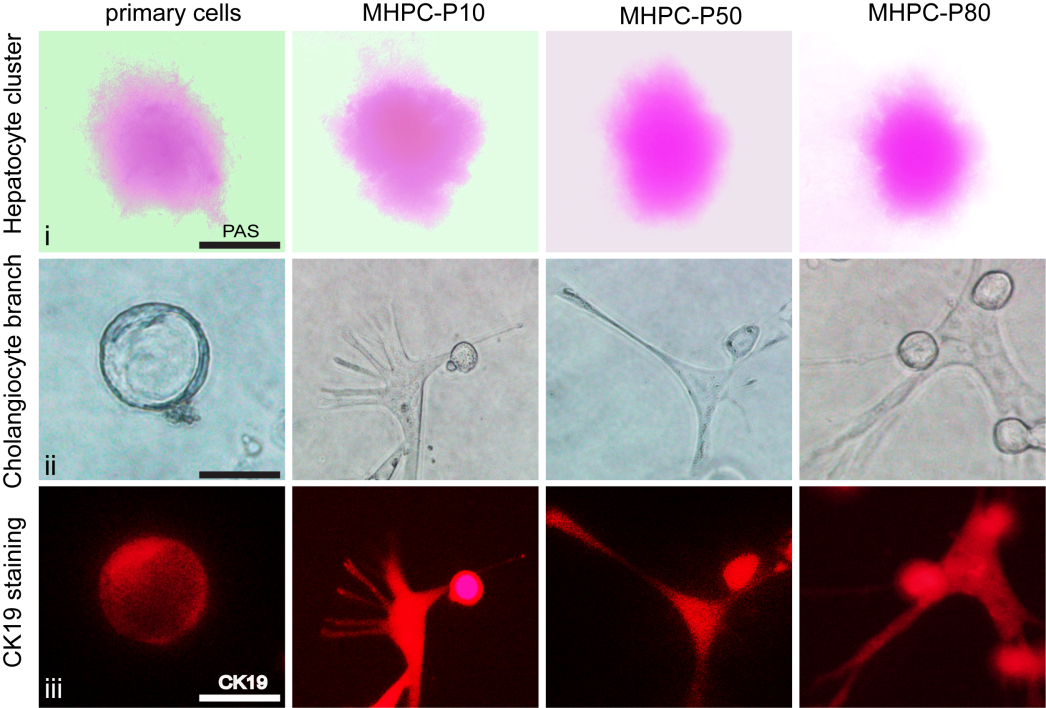
a**

**
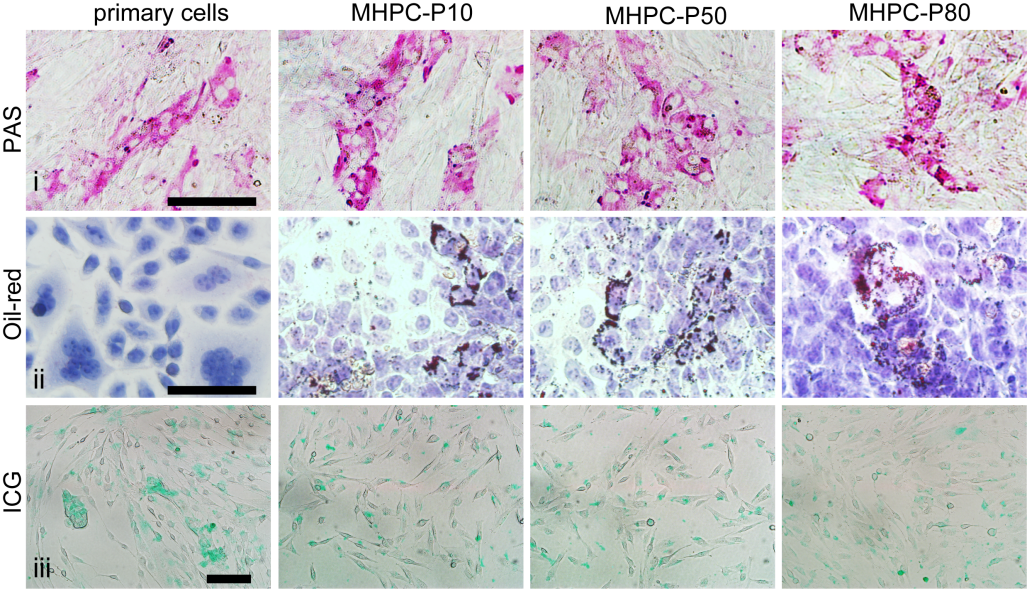
b**

**Supplementary Figure 3. In vitro evaluation for bipotency of different passages of MHPCs and isolated primary cells.** (**a**) *In vitro* bipotency of different passage of MHPCs including (i) PAS staining for glycogen storage in MHPC-derived hepatocytes with 20 ng/ml OSM induction on 2D matrigel, and (ii) (ii) branching structure of cholangiocytes formed by culturing in 3D type 1 collagen gel culture system, and (iii) CK 19 staining (scale bar = 100 µm). (**b**) *In vitro* functional evaluation of MHPCs derived hepatocytes including (i) PAS staining for glycogen storage; and (ii) Oil red O staining for lipid accumulation and (iii) Indocyanine green (ICG) uptake.

**Supplementary Figure 4**

**
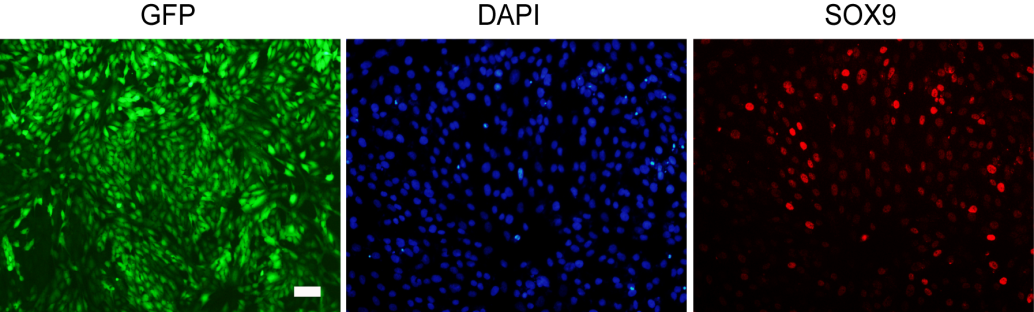
a**

**
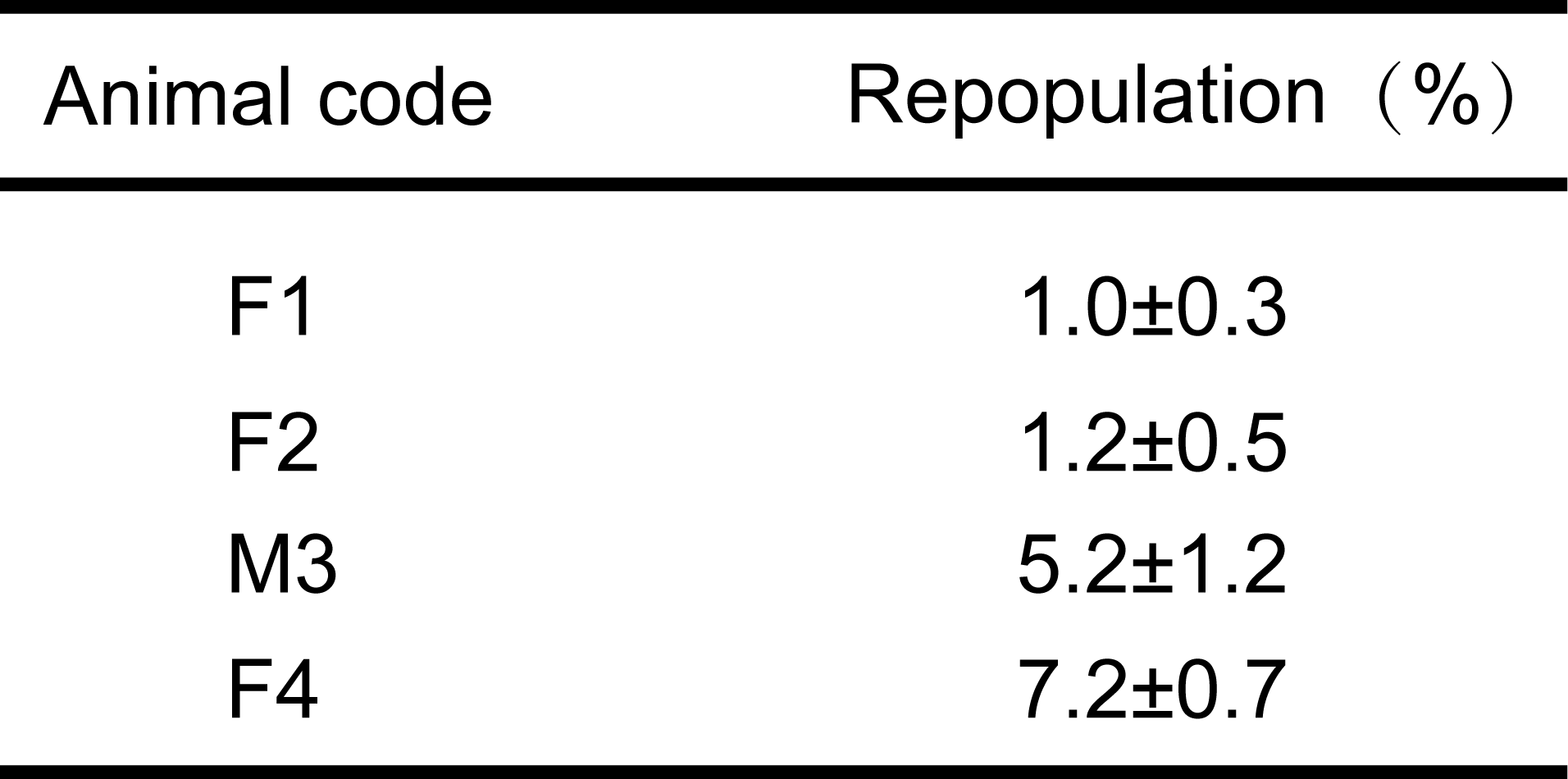
b**

**
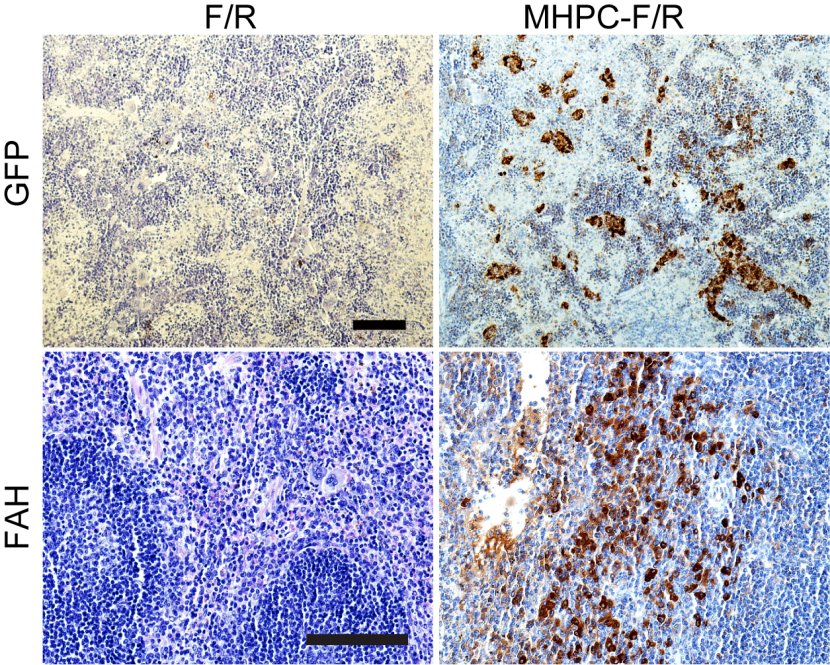
c**

**Supplementary Figure 4. Characterization of EGFP-positive MHPCs and immunostaining of FAH and GFP in spleen tissues from transplanted *F/R* mice.** (**a**) Immunocytochemistry of hepatic progenitor cell marker –SOX 9 in EGFP-positive MHPCs (passage 30) (scale bar=100 µm). Nuclei were counterstained with DAPI. (**b**) Quantitative analysis of Fah-positive cells in total hepatocytes in liver tissues from MHPC-transplanted *Fah^-/-^* mice (n=4). F1 (female), F2, M3 (male) or F4 refers to animal code. (**c**) Immunostaing of FAH and GFP in spleen tissues from transplanted *F/R* mice (scale bar=100 µm).

**Supplementary Figure 5**


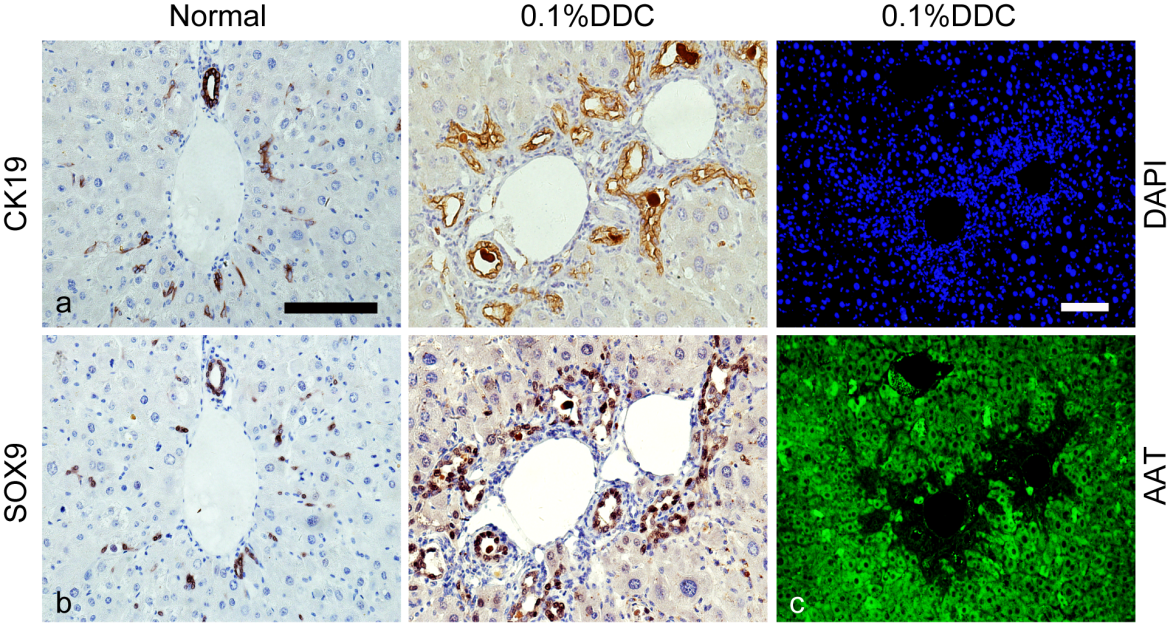


**Supplementary Figure 5. Immunostaining of CK19 (a), SOX9 (b) and AAT (c) in liver tissues from transplanted DDC-injured nude mice.** The results indicated that SOX9 positive progenitor cells mostly differentiated into CK19 positive cholangiocytes, while these cells did not differentiate into AAT positive hepatocytes (scale bar=100 µm).
